# High IQ in Early Adulthood Is Associated with Parkinson’s Disease

**DOI:** 10.3233/JPD-202050

**Published:** 2020-10-27

**Authors:** Camilla Fardell, Kjell Torén, Linus Schiöler, Hans Nissbrandt, Maria Åberg

**Affiliations:** a Department of Pharmacology, Institute of Neuroscience and Physiology, The Sahlgrenska Academy, University of Gothenburg, Gothenburg, Sweden; b Section of Occupational and Environmental Medicine, School of Public Health and Community Medicine, Institute of Medicine, The Sahlgrenska Academy, University of Gothenburg, Gothenburg, Sweden; c School of Public Health and Community Medicine/Primary Health Care, Institute of Medicine, The Sahlgrenska Academy, University of Gothenburg, Gothenburg, Sweden; d Region Västra Götaland, Regionhälsan, Gothenburg, Sweden

**Keywords:** Parkinson’s disease, IQ, cognition, smoking, education

## Abstract

**Background:**

High education level and high occupational complexity have been implicated as risk factors for Parkinson’s disease (PD).

**Objective:**

The objective was to determine whether cognitive capacity, measured as IQ, in early adulthood is associated with the subsequent development of PD.

**Method:**

Data on IQ were retrieved from the Swedish Military Service Conscription Registry, comprising Swedish males who enlisted for military service in the period 1968–1993 (N = 1,319,235). After exclusion, 1,189,134 subjects in total were included in the present study. Individuals who later developed PD (N = 1,724) were identified using the Swedish National Patient Register and the Swedish Cause of Death Register.

**Results:**

High education level was associated with PD. High IQ was associated with PD (*p* < 0.0001), both when analyzed as a continuous variable and when divided into three categories. The hazard ratio for the high IQ category compared to the low IQ category was 1.35 (95% confidence interval 1.17–1.55). Strong test results on the subtests, measuring verbal, logic, visuospatial and technical abilities, were also associated with PD. In a subgroup, smoking was inversely associated with PD, as well as with IQ.

**Conclusions:**

This study identifies high IQ to be a risk factor for PD.

## INTRODUCTION

It has been reported that high level of education is a risk factor for Parkinson’s disease (PD) [1, 2]. Furthermore, in a twins study, PD was more common in individuals who engaged in work of high complexity involving data and people [3].

Many studies have identified associations between PD and specific occupations, for example teaching and healthcare services, clerkship, social science, law and library jobs, and farming, many but not all of which require a high level of education [4–7]. In contrast, the performance of outdoor work has been shown to be inversely associated with PD [8, 9]. Most of the discussions regarding associations between PD and education and occupations have focused on the possibility that certain occupations may increase the risk for PD by exposure to environmental factors, such as toxins (e.g., in farming) or infections (e.g., in teaching and healthcare services).

However, since IQ is a strong predictor of time spent in education and later job status, cognitive performance might be part of the etiology for the association between high-level education and PD. To test this hypothesis, data on cognitive capacity were retrieved from the Swedish Military Service Conscription Registry, which comprises Swedish males who enlisted for military service in the period 1968–1993 (N = 1,319,235). Individuals who later developed PD (N = 1,724) were identified in the Swedish National Hospital Discharge Register.

## MATERIALS AND METHODS

### Study population

**Fig. 1:**
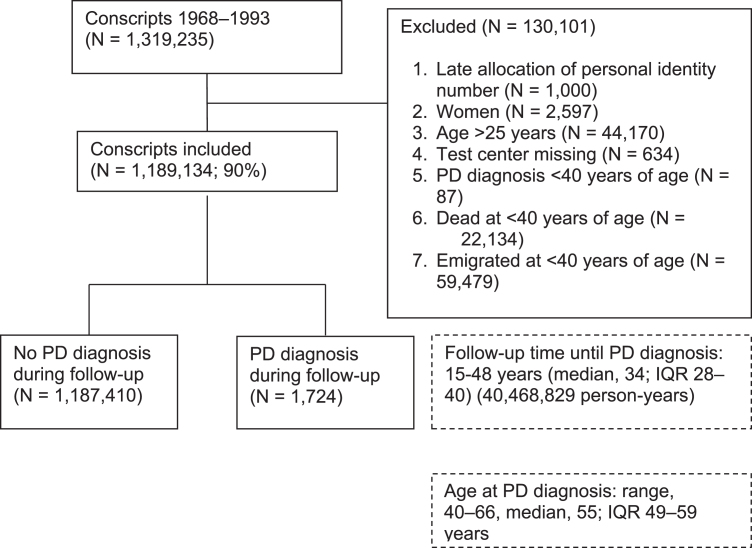
Flow chart illustrating the exclusion criteria, number of diagnoses of Parkinson’s disease (PD), and follow-up time of the Swedish male conscript study population in the period 1968-1993, based on the recommendations in Strengthening the Reporting of Observational Studies in Epidemiology (STROBE) [38].

We performed an exploratory, population-based, prospective study of late-adolescent men who underwent compulsory military conscription examinations. Every Swedish resident is assigned a unique personal identification number, making linkage between different registers possible. The present study is based on data retrieved from the Swedish Military Service Conscription Registry, comprising all Swedish males who were born in the period of 1949–1975, who enlisted for military service in the period of 1968–1993 (N = 1,319,235). During that period, military conscription was compulsory and only 2% –3% of all Swedish men were exempted from conscription, in most cases due to severe mental or physical disorders, or imprisonment. Among the subjects who developed PD during the follow-up, only those who were diagnosed at or after the age of 40 years were included in the present study. The exclusion criteria were: late allocated personal identity number (i.e., personal identity number reallocated from deceased person), female sex, >25 years of age at conscription, missing data regarding the conscription test center, or PD diagnosis, death or emigration before 40 years of age. The remaining 1,189,134 subjects included in the present study were born in the period 1949–1975 and enlisted for military service in the period 1968–1993 (Fig. 1). In total, 1,724 of the included subjects developed PD later in life.

### Conscription register data

The conscripts underwent standardized physical and cognitive examinations conducted by a psychologist and a physician at one of six conscription centers in Sweden (Southern, Western, Eastern, Central, Northern Lower, and Northern Upper).

#### IQ measurements

The cognitive abilities were assessed at conscription using the Swedish Enlistment Battery (SEB). Two different versions of the SEB were used during the study period: SEB67 and SEB80. They consisted of four subtests that measured verbal, logical, visuospatial, and technical capabilities, the scores for which were then summed to give a global IQ score. The outcome of each subtest and the global IQ score were ranked on a scale of 1–9, with 1 being the lowest scoring group and 9 the highest scoring group. The SEB67 battery was used during the period of 1968–1979. “Instructions” was a logical test comprising 40 items that measured the ability to understand instructions and apply them to solve a problem. “Concept Discrimination” consisted of 40 items and examined verbal ability by having the conscript choose which word out of five alternatives did not agree with the others conceptually. “Paper Form Board” was a visuospatial test with 25 items, containing questions about two-dimensional puzzles. “Technical Comprehension” was a 52-item test comprising illustrated technical and physical problems.

The SEB80 battery was used during the period 1980–1993. “Instructions” was retained with some improvements. “Concept Discrimination” was replaced by “Synonyms”, which involved 40 items to assessing vocabulary by letting the conscript identify which out of four alternatives was the synonym of a given word. “Paper Form Board” was replaced by another visuospatial test called “Metal Folding”. This 40-item test measured geometrical perception by having the conscript identify the correct three-dimensional object from a series of two-dimensional drawings. “Technical Comprehension” was modified to measure knowledge of mathematics, physics and chemistry, consisting of 40 items.

#### The 1969–1970 cohort

This sub-cohort consisted of 49,321 Swedish males, born in the period 1949–1951, who were conscripted for compulsory military service in the period 1969–1970. Apart from the standard physical and cognitive examination, the 1969–1970 conscripts participated in a survey designed to collect information on social background, behavior and social adjustment, and substance use, e.g., alcohol consumption and tobacco smoking. The conscripts were asked to report their smoking habits according to one of the following five levels: non-smoker, 1–5 cigarettes per day, 6–10 cigarettes per day, 11–20 cigarettes per day, and >20 cigarettes per day.

#### Educational level

Information on the conscripts’ levels of education and those of their parents were obtained from the Longitudinal Integration database for Health Insurance and Labour Market Studies (LISA) [40]. This database, which was started in 1990 and includes all registered residents aged 16 years and older, integrates data from the labor market and educational and social sectors. Every 5 years between 1960 and 1990, the government agency Statistics Sweden sent out a questionnaire to collect data on variables such as age, sex, civil status, country of birth, citizenship, education, employment, and occupation from all Swedish adults aged ≥16 years. Swedish law stipulated that these surveys had to be completed and this census had a response rate of over 98%. Most data in LISA from 1990 have other sources and are not self-reported; most of the variables are collected automatically from schools and institutions. There is a file for every individual which is updated every year, containing the highest educational level attained. In the analysis, education was classified into three categories (educational levels): pre-high school up to 9 years (low); high school up to 12 years (medium); and university ≥2 years and postgraduate (high). Each category was divided in groups based on the period in which the subject was conscripted: conscription before 1975, in the period 1975–1984, and in the period 1985–1993. For the parents of the conscripts, the highest level of education attained by either parent was used.

### Outcomes

#### Data sources

Data from the Swedish Military Service Conscription Register were linked to the Swedish National Patient Register (NPR) and the Swedish Cause of Death Register. The Swedish National Inpatient Register (IPR), also called the Swedish Hospital Discharge Register, is a part of the NPR and was initiated in 1968. The coverage of IPR gradually increased until 1986 and has complete national coverage since 1987. Since 2001, NPR also contains information on out-patient visits. Deaths were identified by linkage with the Swedish Cause of Death Register, which is updated annually based on the issuance of death certificates and covers virtually all deaths since 1961.

#### PD diagnoses

PD diagnoses in the study cohort through December 31, 2016, were acquired using the conscripts’ personal identification numbers from the NPR or the Swedish Cause of Death Register. From 1987 to 1996, the International Classification of Diseases, ninth and tenth revisions (ICD-9 and ICD-10) were in use. Diagnoses of PD were coded as 332A in records using the Swedish version of the ICD-9 (1987–1997) and as G20 in records using the ICD-10 (1998–2012). The NPR is administered by the Center for Epidemiology of Sweden’s National Board of Health and Welfare. Diagnoses recorded in the NPR have shown a high degree of validity, with positive predictive values of 85% –97% [39].

### Statistical analysis

The follow-up period started at the date of conscription (baseline) and subjects were followed until the time of: 1) first hospitalization for PD or hospital-based outpatient clinic contact for PD; 2) death; 3) emigration; or 4) the end of follow-up, on December 31, 2016 (follow-up: minimum, 15 years; maximum, 48 years). Cox proportional hazards models were used to assess the influences of presumptive predictors on PD diagnosis. Tests based on scaled Schoenfeld residuals showed no significant violations of the proportional hazards assumption.

Intelligence scores were scaled on a nine-point standard scale (stanine = standard nine) and then divided into three groups: low (stanine score 1–3); medium (4–6); and high (7–9), with the latter used as the reference. Education and parental education was trichotomized (low, medium, and high), as described earlier. Conscription year and test center were adjusted for in all the regression models, to account for temporal differences and procedural differences across the participating sites. A cubic restricted spline with knots at the 5th, 35th, 65th and 95th percentiles was used for conscription year. Furthermore, age at conscription was adjusted so as to be linear due to the limited range. The statistical calculations were performed with the SAS ver. 9.4 software (SAS Institute, Cary, NC).

The Ethics Committee of the University of Gothenburg and Confidentiality Clearance at Statistics Sweden approved the study. The requirement for informed consent was waived for the current study as it entails a secondary analysis of existing data. The investigation conforms to the principles outlined in the Declaration of Helsinki in relation to ethical principles for medical research involving human subjects.

## RESULTS

Of the 1,319,235 conscripts in the present study, 1,724 were diagnosed with PD during the 48 years of follow-up (Fig. 1). Almost all cases (N = 1718) were identified by the NPR, of which 1561 were identified by the outpatient hospital register and 157 by the inpatient hospital register. A total of 6 cases were identified by the Cause of death register. In total, 130,101 conscripts were excluded from the analyses. Overall, 40,468,829 person-years of follow-up were included in the study. The observed median age at PD diagnosis was 55 years (range, 40–66 years).

The distributions of educational levels of the conscripts and of their parents are shown in Table 1. Parental education and conscript education was correlated (*r* = 0.4, *p* < 0.0001) as well as parental education and conscript IQ (*r* = 0.5 *p* < 0.0001). We evaluated the education levels of the conscripts and their parents, smoking histories, and IQ levels in relation to the risk of developing PD. Cox proportional hazards models show that education was prospectively associated with PD in analyses adjusted for age, conscription year and conscription test center with HR 1.2 (95% CI 1.01–1.32) for high school and HR 1.4 (95% CI 1.19–1-58) for University and postgraduate (both compared to pre-high school). Corresponding Cox regressions for the highest parental educational level showed no statistically significant differences. When considered as a continuous variable, Global IQ and all four subtests of IQ taken individually were associated with PD (Table 2). High scores on IQ tests were associated with an increased risk of being diagnosed with PD later in life. When dividing the test scores into three categories (1–3, 4–6, 7–9), high Global IQ at conscription was associated with a 1.3- fold (95% CI 1.17–1.55) increase in risk for PD later in life, as compared to low Global IQ. High test scores on the three subtests that measured logical, verbal, and technical IQ levels were associated with 1.2-fold (95% CI 1.02–1.37), 1.3- fold (95% CI 1.15–1.55), and 1.3-fold (95% CI 1.09–1.45) increased risk for PD, respectively. In the analyses that adjusted for age at conscription and birth year, higher IQ level was associated with an increase in the risk for PD. Parental education level did not affect the results.

**Table 1:** Distribution and percentages of male conscripts by their IQ, their educational level and the educational level of their parents

	*Conscripts’ education*			*Parental education*		
	IQ 1–3	IQ 4–6	IQ 7–9	IQ 1–3	IQ 4–6	IQ 7–9
	(N = 235,831)	(N = 620,232)	(N = 278,339)	(N = 234,103)	(N = 617,063)	(N = 276,705)
Pre-high school (≤9 years)
Conscription<1975	22,890 (47.1%)	31,437 (24.3%)	5,244 (7.8%)	33,101 (69.7%)	72,016 (56.8%)	26,847 (40.5%)
Conscription 1975–1984	31,483 (33.9%)	36,748 (15.1%)	4,458 (4.2%)	49,671 (53.6%)	95,537 (39.3%)	25,950 (24.4%)
Conscription 1985–1993	19,499 (20.7%)	14,945 (6.0%)	1,449 (1.4%)	32,870 (35.0%)	52,810 (21.4%)	11,425 (11.0%)
High school (≤12 years)
Conscription before 1975	23,133 (47.6%)	67,530 (52.3%)	23,576 (34.8%)	12,394 (26.1%)	42,328 (33.4%)	24,246 (36.5%)
Conscription 1975–1984	56,966 (61.3%)	149,024 (61.1%)	34,676 (32.6%)	35,999 (38.9%)	104,784 (43.1%)	40,986 (38.6%)
Conscription 1985–1993	67,918 (72.1%)	151,627 (61.4%)	24,453 (23.5%)	49,835 (53.0%)	126,180 (51.1%)	40,487 (38.9%)
University (≥2 years)
and postgraduate
Conscription before 1975	2,624 (5.4%)	30,265 (23.4%)	38,839 (57.4%)	1,979 (4.2%)	12,408 (9.8%)	15,261 (23.0%)
Conscription 1975–1984	4,509 (4.9%)	58,199 (23.9%)	67,387 (63.3%)	6,938 (7.5%)	43,079 (17.7%)	39,299 (37.0%)
Conscription 1985–1993	6,809 (7.2%)	80,457 (32.6%)	78,257 (75.1%)	11,316 (12.0%)	67,921 (27.5%)	52,204 (50.1%)

All percentages are column percentages by each conscription time period. IQ groups: low (1–3); medium (4–6); and high (7–9).

**Table 2:** Hazard ratios for Parkinson’s disease in relation to IQ, education and parental education

	Hazard ratio	95% CI	*p-*value
**A**
Model 1:
Global IQ	1.05	1.03–1.08	<.0001
Subtest 1: Logical IQ	1.04	1.01–1.06	0.008
Subtest 2: Verbal IQ	1.05	1.03–1.08	0.0001
Subest 3: Visuospatial IQ	1.04	1.01–1.07	0.005
Subtest 4: Technical IQ	1.04	1.02–1.07	0.002
**B**
Model 1:
Global IQ	1.35	1.17–1.55	<.0001
Subest 1: Logical IQ	1.18	1.02–1.37	0.03
Subest 2: Verbal IQ	1.34	1.15–1.55	0.0001
Subtest 3: Visuospatial IQ	1.15	0.99–1.34	0.07
Subtest 4: Technical IQ	1.26	1.09–1.45	0.001
Model 2:
Global IQ	1.22	1.03–1.43	0.02
Model 3:
Global IQ	1.37	1.18–1.60	<.0001
**C**
Education	1.37	1.19–1.58	<.0001
**D**
Parental education	1.06	0.92–1.23	0.4

A) IQ test results were used as a continuous variable in Model 1, with baseline adjustments (conscription year, age at conscription and test center). B) Intelligence scores were collapsed into three groups, whereby the lowest scoring group was compared to the highest scoring group. The scores from the four subtests were summed to derive the Global IQ value. Model 1 is analyzed with baseline adjustments (conscription year, age at conscription and test center). Model 2 has baseline adjustments plus adjustments for the conscript’s education. Model 3 has baseline adjustments plus adjustments for the conscript’s parental education.

The descriptive data regarding smoking retrieved from the 1969–1970 sub-cohort is shown in Table 3. Of the 49,571 men evaluated at conscription during 1969 and 1970, there were 256 individuals who later received a diagnosis of PD. The PD cases reported smoking significantly fewer cigarettes at conscription than did the non-PD individuals. Table 4 presents descriptive information about IQ and smoking. Smoking prevalence significantly decreased as Global IQ test scores increased (*p* < 0.0001), with a Spearman correlation coefficient of –0.15. Fifty-two percent of conscripts with high Global IQ test scores (stanine 7–9) reported to be non-smokers, whereas 32% of the conscripts with low Global IQ test score (stanine 1–3) reported to be non-smokers. In the 1969–1970 sub-cohort, high IQ (compared to low) at conscription was not associated with later diagnosis of PD, HR 1.16 (95% CI 0.82–1.64) without adjustments and 1.05 (95% CI 0.73–1.50) with adjustment for smoking.

**Table 3:** Smoking habits at conscription and risk for Parkinson’s disease (PD)

	Individuals (N = 49,514)	Incidence rate per 100,000 person-years	Hazard ratio	95% CI	*p*-value
No smoking	20,496	15.2	Reference
1–5 cigarettes/day	5,566	9.7	0.66	0.43–1.00	0.05
6–10 cigarettes/day	10,288	8.0	0.52	0.36–0.75	0.0005
11–20 cigarettes/day	11,373	9.8	0.64	0.46–0.89	0.007
>20 cigarettes/day	1,791	7.5	0.49	0.22–1.12	0.09

Data from an investigation of smoking habits of men enlisted for military service in 1969 and 1970.

**Table 4:** Smoking habits and global intelligence levels of the conscripts

IQ	>20 cig./day	11–20 cig./day	6–10 cig./day	1–5 cig./day	No smoking
Low	510 (5.5%)	2,413 (25.7%)	2,319 (24.7%)	1,163 (12.4%)	2,974 (31.7%)
Average	900 (3.7%)	6,135 (25.1%)	5,418 (22.1%)	2,704 (11.0%)	9,314 (38.1%)
High	381 (2.4%)	2,825 (18.0%)	2,551 (16.3%)	1,699 (10.9%)	8,208 (52.4%)

Data from an investigation of smoking habits of men enlisted for military service in 1969 and 1970. The correlation coefficient is 0.15 (Spearman), *p* < 0.0001.

## DISCUSSION

The main finding of the present study is that a high IQ is associated with an increased risk for PD (Table 2). We confirm the previously reported association between high education level and PD. The association between a high level of education and PD could be due to a high IQ level. It is well-established that high IQ and education level are correlated, at least in later generations in which education is not so dependent upon parental socioeconomic factors.

To the best of our knowledge, this is the first study to report the association of high IQ and the risk of PD. More usually the opposite is the case: low education level, low IQ, and low socioeconomic status are associated with disease. Low IQ scores have been associated with cardiovascular events and disease, suicide, excessive alcohol consumption, and schizophrenia [10–19]. In line with the hypothesis that higher intelligence affects health outcomes through superior ability to prevent and manage disease, those with higher intelligence should be at lower risk. However, in the case of PD, we found the opposite.

Since IQ is to a major extent inherited [20] we investigated the levels of education of the parents of our subjects. Whereas, no information is available on parental IQ levels, there is information on parental education levels, which can be considered as a proxy of socioeconomic level and childhood environment. To our surprise, there was no significant association between parental education level and the presence of PD of the conscript. A probable explanation for this lack of association is that for most of the conscripts who later developed PD, their parents were born in the 1920s and 1930s. A high level of education was still quite rare at that time, which is why education is not a good reflection on IQ for those individuals, because it is more likely to reflect socioeconomic status than IQ or a person’s ability to attain a high education.

Previous studies have shown that individuals with low cognitive ability in early life are more likely to become smokers [21], less likely to stop smoking [22], and drink more alcohol [23] than individuals with higher cognitive ability. Since smoking shows a robust negative association with PD [24–28], we investigated the associations between smoking and PD and IQ in a sub-cohort of the conscripts. We confirm the negative association of smoking with PD (Table 3). In addition, we show (Table 4) an inverse association of smoking with IQ. However, IQ at conscription was not associated with later diagnosis of PD. Due to the low number of PD cases in the 1969–1970 sub-cohort (*n* = 256), there is a lack of power and the precision of this analysis is limited.

It has been discussed extensively whether the association between smoking and PD is due to cytoprotection or reversed causality, in the sense that smokers are more likely to quit smoking during prodromal PD symptoms or that the prodromal PD personality tends to choose not to smoke [25, 26]. Many experimental animal studies have suggested that nicotine is cytoprotective, while other substances present in cigarette smoke have also been proposed to be cytoprotective [29]. Also, a study on twins suggests a protective effect of smoking [41]. However, several findings speak against cytoprotection as a causal factor for the association with PD. One is that the rate of progression of symptoms is not modified by smoking in patients with PD [30–32]. Another aspect is that in a large prospective study, no difference was observed for the age at onset of PD between ever-smokers and never-smokers [33]. This was also reported in a study on moist smokeless tobacco [34]. Finally, and most compelling, is the finding that nicotine patch treatment did not slow the progression of PD symptoms in humans [35, 36]. It is possible that smoking is a proxy of some other factor associated with IQ. In another twin study, the protective association of smoking is less-pronounced, indicating that some underlying genetic or environmental factors are of importance [37]. IQ itself would probably also influence the individual’s exposure of environmental factors, such as infections, toxins, trauma etcetera during life, all factors that have been suggested to be of importance for the etiology of PD (for review see Wirdefeldt et al. [27]). The IQ level of an individual may influence the person’s occupation and to which extent the person has a sedentary lifestyle.

The unique strength of the present study is the use of a nationwide cohort, with a study sample consisting of 1,319,235 conscripts, the vast majority of all Swedish men born between 1951 and 1965. A sample size this large allows for a more accurate estimate of the association between IQ scores and PD risk. Having a study sample that represents such a large percentage of the population also eliminates the risk for selection bias. Many studies on early-life factors and later health outcomes rely on retrospective information collected at some point of the subject’s adult life which may introduce bias [42].

A limitation of the present study is that it included only men, thus, our results may not apply to women. Another potential limitation is that the diagnosis of PD may be better in highly educated people, which could affect the associations. Furthermore, although men in our study were followed for a maximum of 48 years, the average age at end of follow-up is relatively young (56 years) and the etiology of these PD diagnoses may differ from those occurring later in life.

In conclusion, the present study shows for the first time an association between high IQ test scores in adolescence and higher risk of being diagnosed with PD later in life. Additional studies on how IQ scores relate to PD risk are required to clarify this further.

## CONFLICT OF INTEREST

The authors have no conflict of interest to report.
